# Adaptations of Bacterial Extracellular Vesicles in Response to Antibiotic Pressure

**DOI:** 10.3390/ijms26115025

**Published:** 2025-05-23

**Authors:** Dell’Annunziata Federica, Ilaria Cosimato, Flora Salzano, Francesca Mensitieri, Vincenzo Andretta, Emanuela Santoro, Giovanni Boccia, Veronica Folliero, Gianluigi Franci

**Affiliations:** 1 Department of Medicine, Surgery and Dentistry “Scuola Medica Salernitana”, University of Salerno, 84081 Baronissi, Italy; federica.dellannunziata@unicampania.it (D.F.); icosimato@unisa.it (I.C.); flsalzano@unisa.it (F.S.); fmensitieri@unisa.it (F.M.); vandretta@unisa.it (V.A.); esantoro@unisa.it (E.S.); gboccia@unisa.it (G.B.); 2 U.O.S Microbiology and Virology, A.O.U San Giovanni di Dio e Ruggi D’Aragona University Hospital, 84126 Salerno, Italy

**Keywords:** extracellular vesicles, outer membrane vesicles, membrane vesicles, Gram-negative bacteria, Gram-positive bacteria, antibiotic resistance

## Abstract

Extracellular vesicles (EVs) are nanometer-sized lipid structures actively secreted by Gram-negative and Gram-positive bacteria, representing a sophisticated microbial adaptation and communication strategy. These structures are involved in biomolecular transport, the regulation of biological processes, the modulation of host–pathogen interactions, and adaptation to hostile environmental conditions. EVs also play a crucial role in virulence, antibiotic resistance, and biofilm formation. This review will explore the biogenesis, composition, and biological mechanisms of outer membrane vesicles (OMVs) secreted by Gram-negative bacteria and membrane vesicles (MVs) generated by Gram-positive bacteria. In detail, the modulation of EVs in response to antibiotic exposure will be addressed. The role of EV morpho-functional adaptations will be studied in antimicrobial resistance, the gene determinant spread, and survival in adverse environments. This study aims to provide a comprehensive overview of the EV role in bacterial physiology, highlighting their ecological, evolutionary, and biotechnological implications. An overview of the enzymes and proteins mainly involved in OMV-mediated resistance mechanisms will also be provided. These insights could open new perspectives for developing therapeutic strategies that counteract EV secretion and biotechnological applications, such as vaccines and drug delivery systems.

## 1. Introduction: Duality of EVs in Gram-Negative and Gram-Positive Bacteria

Bacterial EVs are nanoscale lipid structures actively secreted by Gram-negative and Gram-positive bacteria [1,2,3]. These vesicles are involved in intercellular transport and communication, loading biomolecules such as proteins, lipids, and nucleic acids into the vesicular lumen [4,5]. EVs fundamentally regulate diverse biological processes, including host–pathogen interaction, virulence, and microbial adaptation, promoting ecological success in complex environments [6,7,8]. The vesicle biogenesis and composition reflect the characteristics of the membrane and cell wall of each bacterial group [9,10]. In Gram-negative bacteria, an outer membrane (OM) forms OMVs [11,12]. These vesicles originate through the curvature of the OM and localized detachment, facilitated by factors such as the accumulation of lipids or asymmetric proteins [13,14].

On the other hand, Gram-positive bacteria lack OM and have a thick and complex cell wall, which poses a challenge for vesicle biogenesis [15,16]. However, recent studies have shown that these bacteria can produce vesicles originating from the cytoplasmic membrane and crossing the peptidoglycan wall [17]. The size of EVs varies depending on species, growth conditions, environmental factors, and genetic determinants. OMVs typically range in size from 20 to 400 nanometers [11]. In contrast, MVs exhibit a broader size distribution, typically ranging from 20 to 500 nanometers [15]. Dimensional variability is also influenced by EV isolation and analysis methods used. Recent advances in label-free and non-invasive techniques, such as optical diffraction tomography (ODT), have enabled the three-dimensional high-resolution imaging of individual EVs, without the need for staining or fixation. ODT provides detailed information on EV morphology and refractive index, supporting both quantitative and qualitative analysis. Furthermore, ODT allows for real-time tracking of EV interactions with target cells, paving new avenues for innovative diagnostic and therapeutic applications [18].The MVs biogenesis in Gram-positive bacteria requires the activity of enzymes that remodel the cell wall, creating temporary openings for vesicle passage [19,20]. Despite these structural differences, both types of vesicles share common functional characteristics. They serve as vehicles for transporting bioactive molecules, such as toxins, proteins, RNA, and DNA, playing fundamental roles in virulence regulation, immune system modulation, and biofilm formation [21,22]. Furthermore, EVs mediate intercellular communication by coordinating collective bacterial behaviors by transferring chemical signals, such as quorum-sensing (QS) molecules [23,24]. The subsequent sections will describe the composition, functions, and biogenesis mechanisms of OMVs secreted by Gram-negative bacteria and MVs produced by Gram-positive bacteria.

### 1.1. OMVs: Composition, Biogenesis, and Functional Roles

**Figure 1:**
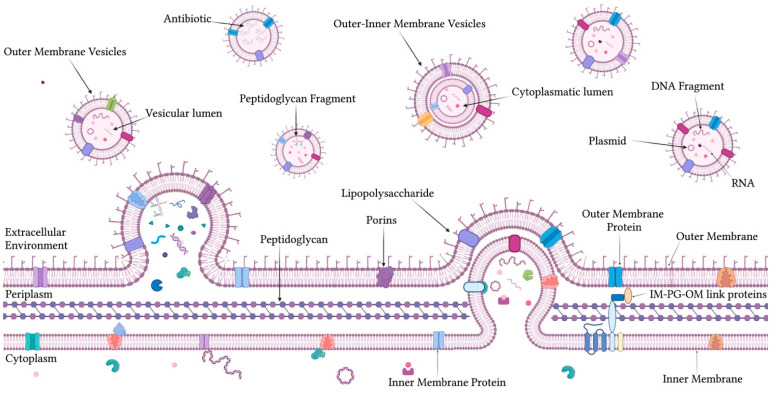
Composition, morphology, and biogenesis of OMVs secreted by Gram-negative bacteria.

The OMV biochemical composition is closely related to the OM of their bacterial origin and varies significantly depending on environmental conditions, bacterial strain, and selective pressures to which the microorganism is subjected [25,26] (Figure 1). OMVs are enriched with outer membrane proteins (Omps), which play a key role in facilitating passive transport and regulating metabolic processes [27,28,29]. OmpA is frequently identified in OMVs and contributes to the structural stability of the membrane and mediates interactions with host cells by acting as an inducer of immune responses [30,31]. OMVs also carry functional proteins, such as iron-binding proteins, which aid in the acquisition of iron from the host environment [32]. Furthermore, OMVs are rich in hydrolytic enzymes, including proteases, lipases, and nucleases, to break down complex substrates and facilitate tissue colonization during infection [33,34]. Many proteins contained in OMVs are bacterial toxins and components of the type V secretion system [35]. These virulence factors directly interact with host cells, altering their physiology and contributing to pathogenic processes [36]. The lipid composition of OMVs mirrors that of the OM, primarily comprising phospholipids and lipopolysaccharides (LPS) [37,38]. Phospholipids in OMVs, such as phosphatidylethanolamine, phosphatidylglycerol, and cardiolipin, constitute the vesicular membrane, influencing its fluidity and curvature [39,40]. LPS is essential for OMV structural integrity and its immunomodulatory activities [41,42]. Lipid A, the toxic component of LPS, is responsible for the immunostimulatory activity of OMVs by acting as an agonist of Toll-like receptor 4 (TLR4) on host immune cells [43,44]. This interaction triggers an acute inflammatory response, which contributes to both host defense and bacterial pathogenesis [45]. A unique feature of OMVs is the presence of genetic material, both DNA and RNA [46]. The latter content consists mainly of messenger RNAs (mRNAs) and non-coding RNAs [47,48]. RNA can modulate gene expression in recipient bacterial cells, acting as mediators of intercellular communication [49,50]. DNA carried by OMVs includes plasmid and genomic fragments that can be transferred to recipient cells through horizontal gene transfer (HGT) mechanisms [46]. This transfer has significant implications for evolutionary adaptation, allowing the spread of genes related to antibiotic resistance, virulence, and environmental adaptability [51]. The vesicular composition is highly dynamic and adjusts to specific extracellular stimuli. For instance, nutrient deficiencies and limited iron availability induce significant changes in the protein and lipid content [52,53]. Similarly, exposure to antibiotics leads to the incorporation of proteins related to antimicrobial resistance and altered LPS, enhancing bacterial survival in challenging conditions [54,55]. Genetic mutations or metabolic alterations can further influence OMV composition, modifying their content and functional impact on both host organisms and other bacteria [56].

OMV biogenesis is a dynamic and regulated process influenced by genetic, environmental, and structural factors. It occurs through two main distinct mechanisms. The blebbing is the predominant pathway for OMV release [57,58]. During this process, specific regions of the OM bend outwards, detaching as spherical vesicles [59]. This curvature and membrane detachment is regulated by molecular factors such as lipid composition, protein content, and membrane tension level [60]. The second mechanism, known as explosive cell lysis, generates hybrid vesicles containing components of both the outer and inner membranes [52,61]. This path occurs following the disruption of the cell wall and the release of intracellular contents, often in response to extreme environmental stresses or antibiotic exposure [62,63]. Several hypotheses have been proposed to explain the details of OMV biogenesis. The first model is based on the reduction of cross-linking between the OM and the underlying peptidoglycan. The weakening of the bond between these structures separates the OM from the peptidoglycan, forming the vesicle [64]. A second model emphasizes the accumulation of specific proteins and lipids in localized regions of the OM. This accumulation generates asymmetric tensions that induce membrane curvature, promoting OMV release [65]. OMPs, such as OmpA and OmpC, as well as short-chain LPS, play crucial roles in this process [66,67]. The accumulation of phospholipids and cardiolipin in specific membrane microdomains contributes to vesicle formation, creating unstable regions that are more easily detached [68,69]. A third model involves the role of LPS in OMV biogenesis. The accumulation of LPS at specific locations on the membrane creates localized surface tension, which promotes budding and vesicle release [70]. OMV biogenesis is tightly regulated at the genetic level. Several genes have been identified as crucial in controlling OMV release, including those encoding membrane-modifying enzymes, structural proteins, and lipid synthesis regulatory genes [71]. Mutations in genes controlling peptidoglycan or lipid metabolism can increase the frequency of vesicle release or modify their composition [72]. Environmental conditions also profoundly influence OMV biogenesis [11,63]. Thermal or osmotic stress, exposure to antibiotics, and the absence of nutrients can alter the vesicular composition, favoring the inclusion of molecules that improve bacterial survival [31,53].

OMVs perform a wide range of biological functions, reflecting their molecular composition, the diversity of the bacterial species and the ecological context in which they are released [73]. These functions include the transport of bioactive molecules, intercellular communication, HGT, modulation of the immune system, and adaptation to environmental stresses [17]. One of the most studied functions of OMVs is their role as vehicles of virulence factors, such as toxins, adhesive proteins, and degradative enzymes [74]. This targeted delivery increases the bacteria virulence impact in the site of infection. Additionally, degradative proteins carried by OMVs, such as proteases or nucleases, facilitate host tissue invasion and manipulation of the local microenvironment, promoting bacterial colonization and infection progression [13,75]. OMVs are involved in the modulation of host immune responses and LPS represents the primary mediator of this interaction [76]. The lipid A component of LPS acts as a potent agonist of TLR4 on innate immune cells, such as macrophages and dendritic cells [77,78]. This interaction can induce an acute inflammatory response characterized by producing pro-inflammatory cytokines, such as TNF-α, IL-6 and IL-1β [79]. However, OMVs can also suppress the immune response by negatively modulating T-cell activation or interfering with antigen presentation [80]. OMVs are key players in intercellular communication in bacteria–bacteria (intraspecific and interspecific communication) and bacteria–host cells [80]. The presence of signaling molecules coordinates bacterial behavior [81]. For instance, in *Pseudomonas aeruginosa (P. aeruginosa)*, OMVs carry QS molecules such as 3-oxo-C12-HSL, which regulate the expression of genes involved in virulence, antibiotic resistance, and biofilm formation [82,83,84]. Furthermore, OMVs can transfer biochemical signals to eukaryotic cells, influencing their physiology and modulating inflammatory or apoptotic responses. An essential function of OMVs is their role in HGT, an important mechanism for bacterial adaptation [85,86,87]. OMVs carry fragments of plasmid and genomic DNA, including genes associated with antibiotic resistance, virulence factors, and other selective adaptations [88]. OMVs also cover the role of bacterial adaptation to environmental stresses [89]. During exposure to oxidative stress or antibiotics, bacteria release OMVs containing potentially harmful molecules, such as oxidizing and hydrolyzing agents, to protect the bacterial cell [90,91]. OMVs can also act as molecular traps, neutralizing toxic compounds or antimicrobial molecules through sequestration within their vesicles [92]. In addition to their biological roles, OMVs have important ecological and applicative implications. OMVs mediate interactions between bacteria and surrounding microbial communities in natural environments, influencing ecological dynamics and population composition [93]. From a biotechnological perspective, OMVs represent a potential delivery system for therapeutic applications [94,95]. Their immunomodulatory properties and ability to deliver bioactive molecules make them promising tools for vaccine development and targeted drug delivery [96,97].

### 1.2. Structure, Biogenesis, and Roles of MVs in Gram-Positive Bacteria

**Figure 2:**
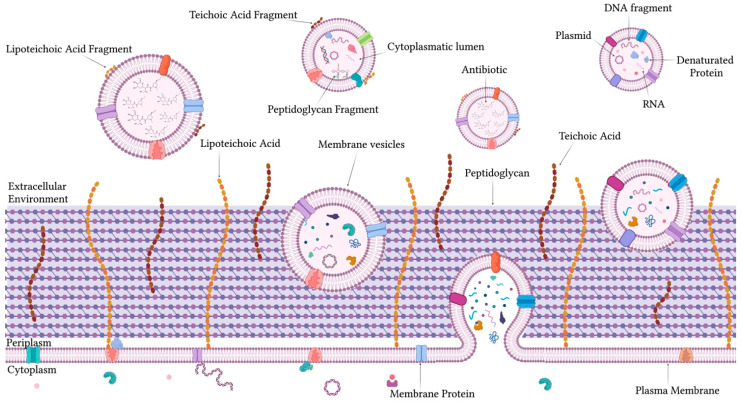
Composition, morphology, and biogenesis of MVs secreted by Gram-positive bacteria.

Proteins present in MVs are derived from the cytoplasmic membrane and cytoplasm, with some incorporated during their passage through the peptidoglycan wall [98,99] (Figure 2). MVs are enriched with hydrolytic and degradative enzymes, such as proteases, nucleases, and glycolases, which enable bacteria to modify their environment, facilitating colonization and invasion [100,101]. Simultaneously, these vesicles transport hemolytic or cytolytic toxins and adhesive proteins, which are delivered in a protected manner directly to target cells, thereby enhancing the efficacy of pathogenic processes [10]. Furthermore, MVs play a fundamental role in resistance to environmental stresses due to the presence of proteins such as chaperonins, GroEL, and DnaK that protect from thermal and oxidative stress. Similarly, proteins associated with the efflux pump contribute to increasing antibiotic resistance, through extracellular expulsion [102,103]. MVs also contain proteins involved in the transport of DNA and RNA, which are for the stabilization and transfer of nucleic acids [104,105]. The protein composition of MVs is not uniform and varies in response to environmental conditions and is closely associated with the characteristics of the cytoplasmic membrane from which they originate [106]. These vesicles contain common phospholipids such as phosphatidylglycerol and cardiolipin [107]. The latter, accumulated in specific regions of the membrane, induces a high curvature [108]. This property facilitates the formation of unstable lipid microdomains, which promote the process of membrane budding. MVs also include glycolipids, lipoproteins, lipid teichoic acids (LTAs), and isoprenoids, which enhance their adaptive functionality [109]. These lipids increase the vesicular stability and improve their interactions with other bacteria or host cells [110]. MVs are also characterized by the presence of nucleic acids, which are protected by the vesicular lipid structure acting as a physical barrier against degradative enzymes [111]. DNA contained in the lumen includes both plasmid and chromosomal fragments [112]. Plasmid, which often encodes genes related to virulence factors, antibiotic resistance, or environmental adaptations, is a critical element for bacterial survival and evolutionary success [113,114]. Chromosomal fragments, randomly or selectively included, contribute to the genetic diversity and adaptation of microbial populations [115,116]. In addition to DNA, MVs harbor a wide range of RNA molecules, including mRNA, sRNA, ribosomal RNA (rRNA), and transfer RNA (tRNA) [117,118]; mRNAs can be transferred to recipient cells and directly translated into proteins, providing new functionalities or metabolic adaptations [119]. Meanwhile, sRNAs cover essential regulatory roles, modulating gene expression in recipient bacteria and influencing processes such as biofilm formation, virulence, and antibiotic resistance [47,120].

MV biogenesis is a complex and highly regulated process, due to the presence of a thick peptidoglycan wall and the absence of an OM [121]. This thick and rigid layer serves as a natural barrier to vesicle formation, but Gram-positive bacteria have evolved adaptive mechanisms to overcome this obstacle and produce functional MVs [122]. These vesicles originate from the cytoplasmic membrane, which bends outward, collecting cytoplasmic and membrane materials [123]. To facilitate the passage of vesicles through the peptidoglycan layer, bacteria employ specific enzymes, such as cell wall hydrolases, including lysozyme and glycohydrolase [124]. These enzymes temporarily remodel the peptidoglycan by creating localized and transient openings that are sufficient to allow vesicular pass without compromising the wall structural integrity [125]. A critical factor in MV biogenesis is the lipid composition of the cytoplasmic membrane. Specialized lipids, such as cardiolipin, accumulate in regions of high membrane curvature, promoting vesicle budding [126]. Furthermore, proteins that regulate membrane tension and dynamics facilitate vesicular separation [127]. External stresses, such as oxidative stress, exposure to antibiotics, or other adverse conditions, can stimulate the release of MVs [53,128,129]. Under these circumstances, the vesicle content can be modulated to include degradative enzymes, toxins, or other factors essential for bacterial survival and interaction with the host [130]. A crucial aspect of the biogenesis process is the gene regulation of enzymes that remodel peptidoglycan [131]. Mutations in genes encoding these enzymes can influence both the frequency and the composition of MVs [132,133].

MVs perform a wide range of crucial roles for bacterial survival, adaptation, and virulence [101]. One of their primary functions is the transport and release of bioactive molecules, including hydrolytic enzymes, toxins, adhesive proteins, and nucleic acids (DNA and RNA) [134]. These molecules facilitate host colonization, tissue invasion, and the manipulation of the surrounding microenvironment [8,135]. For instance, proteases and nucleases contained in MVs can degrade host extracellular barriers, such as neutrophil extracellular traps (NETs), while hemolytic or cytolytic toxins disrupt host cell membranes, aiding bacterial dissemination [136,137]. MVs also modulate the host immune system through the release of LTAs and regulatory RNAs, allowing bacterial persistence within the host [18,138,139]. Furthermore, these vesicles are essential for intercellular communication by transporting molecular signals such as regulatory RNAs and QS proteins, coordinating collective bacterial behaviors, including biofilm formation and virulence regulation [140,141,142]. Under conditions of environmental stress, MVs act as “molecular traps”, sequestering toxic compounds or distributing stress-resistance proteins, thereby protecting the bacterial cell [143,144]. Furthermore, MVs facilitate the spread of genetic material through HGT, promoting adaptation and the dissemination of resistance or virulence genes within microbial communities [145,146]. These diverse functional roles underscore the importance of MVs as an essential tool for the ecological and evolutionary success of Gram-positive bacteria [15].

## 2. Modulation of Bacterial Vesicles in Response to Antibiotics

Antibiotic exposure represents one of the strongest selective pressures on bacteria, driving the development of adaptive strategies. A key mechanism in this adaptation involves the morpho-functional modulation of secreted EVs, which play a crucial role in bacterial survival. In response to antibiotics, these vesicles undergo structural and molecular modifications that enhance the ability to protect bacterial cells and interact with their surroundings [114]. For instance, antibiotic exposure can increase the EVs production enriched with degradative enzymes capable of inactivating antibiotics. Simultaneously, EV composition can be modified to incorporate resistance-associated molecules, such as efflux pump effectors and stress response proteins. Vesicles may exhibit changes in membrane curvature and stability, enhancing their capacity to transport protective or detoxifying factors. Moreover, OMVs and MVs can sequester antibiotics within their lumen, functioning as molecular “decoys” that reduce the direct exposure of bacterial cells to antimicrobial agents. This process protects bacteria from the effects of antibiotics and favors the spread of resistance genes through HGT, amplifying the adaptability of microbial populations [123]. The following sections will provide an in-depth review of the evidence on the complex mechanisms through which bacteria modulate the structure, composition, and functionality of vesicles in response to antibiotic exposure. Morphological modifications, enrichment with bioactive molecules, the modulation of resistance proteins, and generally adaptive strategies employed to overcome the burden induced by antimicrobial agents will be examined [123]. This review will also highlight the molecular processes regulating vesicle biogenesis under these conditions and discuss the ecological and evolutionary implications of these adaptations for bacterial populations.

### 2.1. Antibiotic-Induced Changes in OMVs Production in Gram-Negative Bacteria

Pioneering studies have shown that antibiotic exposure can significantly influence the quantity, structure, and protein content of OMVs, altering their biological functions and virulent potential (Table 1). Moreover, antibiotic exposure stimulates OMV production through mechanisms destabilizing the OM, activating the SOS response and modifying bacterial gene expression. They also influence intercellular communication, antibiotic resistance, and biofilm formation, highlighting their crucial role in bacterial adaptation and the evolution of antimicrobial resistance.

#### 2.1.1. Secretion of *P. aeruginosa*-OMVs Under Antibiotic Stress

Kadurugamuwa and Beveridge were the first to investigate the effects of gentamicin on the production and composition of OMVs secreted by *P. aeruginosa.* Under physiological growth conditions, *P. aeruginosa* spontaneously releases vesicles; however, exposure to gentamicin significantly increased vesicle production. Indeed, the TEM analysis revealed an increase in “bleb” formation on the cell surface of gentamicin-treated bacteria, resulting in vesicles slightly larger than those naturally produced (100 nm vs. 80 nm). A nano-tracking analysis demonstrated a threefold increase in vesicle production in response to treatment, attributed to OM destabilization. Biochemical analyses via SDS-PAGE revealed partial differences in the protein composition of natural OMVs (n-OMVs) and gentamicin-induced OMVs (g-OMVs). Notably, g-OMVs contained a ~35 kDa protein absent in n-OMVs. Immunoblotting and immunogold microscopy revealed that both vesicle types included LPS, but g-OMVs showed significant enrichment in type B LPS. These vesicles exhibited enzymatic activities. Phospholipase C (PLC), alkaline phosphatase, and several proteases were concentrated in the vesicles, while elastase was detected exclusively in the supernatant. Enzymatic activities were confirmed through biochemical assays and immunolocalization, indicating that PLC and alkaline phosphatase are initially localized in the cytoplasm or cell membrane before their release via vesicles. Gentamicin-induced vesicles showed higher enzymatic activities, correlated with increased vesicle production. Additionally, DNA was detected within vesicles at higher concentrations in g-OMVs than in n-OMVs. These findings proposed that OMVs serve as a secretion mechanism for virulence factors and other cellular components during infection, facilitating their delivery to target tissues. Gentamicin, by perturbing the OM, amplifies vesicle release, enriching it with proteins, enzymes, and DNA [147]. These first results have laid the foundation for further investigations, deepening the role of antibiotics in the morpho-functional modulation of OMVs. Nearly 20 years later, new evidence showed that ciprofloxacin treatment induces a significant increase in *P. aeruginosa*-OMV production, through activation of the SOS response. Treatment with ciprofloxacin (1 µg/mL) caused marked cell lysis in wild-type bacteria compared to the lexAN mutant, which is unable to activate the SOS response. Protein content in OMVs from treated wild-type bacteria increased over 100-fold compared to untreated controls. In contrast, OMV production in the lexAN mutant bacteria increased only 33% compared to the wild-type strain. These findings could indicate that the SOS response plays a crucial role in stimulating OMV production. These findings indicate that the SOS response plays a crucial role in stimulating OMV production. Functionally, OMVs from ciprofloxacin-treated wild-type bacteria exhibited enhanced cytotoxicity toward macrophages, resulting in a 55% increase in lactate dehydrogenase release compared to untreated controls. This cytotoxic effect was abolished in OMVs from lexAN mutant bacteria, further underscoring the role of the SOS response in amplifying OMV toxicity. A proteomic OMVs analysis identified 145 proteins, categorized into three groups: 74 SOS-related proteins (detected only in treated wild-type bacteria), 35 common proteins (SOS-independent), and 36 specifics to lexAN bacteria. SOS-related proteins included FtsK and catalase, associated with antibiotic resistance and virulence, as well as proteins involved in cell motility and efflux. Notably, metalloproteases linked to tissue penetration and pathogenicity were exclusively associated with SOS-related proteins. These findings demonstrated that ciprofloxacin-induced activation of the SOS response amplifies OMV production and alters their protein composition, enhancing bacterial survival and virulence. This highlights the need to understand the interaction between antibiotic stress and bacterial pathogenicity, to guide the development of more effective antimicrobial therapies [148]. A new development from previous studies evaluated the combination therapy of meropenem and ciprofloxacin (MCC) to influence OMV production and virulence in multidrug-resistant (MDR) *P. aeruginosa*. Clinical isolates were collected from a Brazilian hospital, which was 53.1% resistant to ciprofloxacin, 15.6% to meropenem, and 31.3% to both antibiotics. Combination tests revealed synergistic activity in 25% of isolates and a reduction in bacterial counts in 40.6% of isolates after 12 h of exposure. However, bactericidal activity was achieved in only 12.5% of isolates. Structurally, MCC treatment induced cellular changes, including cell rounding, cell wall disintegration, and a reduction in OMV production. Scanning electron microscopy (SEM) and TEM revealed that MCC treatment significantly decreased OMV production compared to monotherapy. After 3 h post-treatment, OMVs were more abundant in cells treated with ciprofloxacin alone, while MCC treatment led to a marked reduction in OMVs at 3 and 12 h. Since OMVs are carriers of virulence factors and contribute to bacterial cytotoxicity, this reduction suggested a favorable effect of MCC in attenuating bacterial virulence. Gene expression analysis further supports this finding: MCC treatment significantly downregulated key genes associated with antimicrobial resistance and cell repair, including ampC, oprD, and lexA, compared to single-drug treatments. These results demonstrated that MCC effectively counteracted the growth of MDR *P. aeruginosa* and decreased host-tissue damage through the reduction of OMV production and modulation of the expression of virulence-related genes [149].

An important perspective on OMVs is their role in regulating intercellular communication, which is crucial for bacterial adaptation to hostile environments. OMVs influence QS and biofilm formation, two key mechanisms essential for the virulence of *P. aeruginosa*. Antibiotic exposure modulates these processes through vesicular effects. In support of this evidence, a study reported that *P. aeruginosa* vesicles enhanced the strain’s resistance to subinhibitory concentrations of polymyxin B, thereby reducing its efficacy. Exposure to 25 and 50 μg/mL OMVs increased the minimum inhibitory concentration (MIC) of polymyxin B by 50% and 150%, respectively. Furthermore, it revealed that the initial bacterial population density influenced the degree of protection conferred by OMVs. Vesicles in low-density populations (10^4^–10^6^ CFU/mL) showed greater protection than in high-density populations (10^7^ CFU/mL). Additionally, polymyxin B at sub-MIC concentrations inhibited the QS system of *P. aeruginosa*, reducing the bacterium’s ability to form biofilms and produce virulence factors (pyocyanin and rhamnolipids).

Although Polymyxin B reduced the overall pathogenicity of the bacterium, it also increased the risk of acquiring resistance through HGT. A mathematical model was developed to quantitatively assess the impact of OMVs on *P. aeruginosa* resistance to polymyxin B. This model showed that OMVs interacted with the antibiotic in the extracellular environment, reducing its efficacy and enhancing bacterial survival. However, the protective effect of OMVs did not increase the intrinsic resistance of *P. aeruginosa* to polymyxin B. OMVs were also found to inhibit the QS system, contributing to a reduction in the ability of *P. aeruginosa* to form biofilms. Indeed, the transcriptomic analysis indicated that the combination of polymyxin B and OMVs altered bacterial gene expression. Genes involved in antimicrobial resistance, communication, and competitive processes were dysregulated, limiting biofilm formation and altering bacterial pathogenicity [150].

#### 2.1.2. Antibiotic-Induced Modulation of *Escherichia coli*-OMVs

The research was extended to other Gram-negative bacteria to understand whether antibiotic exposure could modulate the composition and functional characteristics of OMVs in other bacterial species. Contextually, Chan and colleagues analyzed OMV production and proteomic variations in two extraintestinal pathogenic *Escherichia coli* (*E. coli)* strains. The bacteria, isolated from the blood of patients with sepsis, exhibited differences in sensitivity to gentamicin, with one strain resistant (GenR) and the other sensitive (GenS). The study focused on OMVs behavior in response to antibiotic-induced stress and iron deficiency. The nanoparticle tracking analysis (NTA) showed that the GenS strain, exposed to 6 µg/mL of gentamicin, exhibited a 13.1-fold increase in vesicle production compared to the control, although the vesicle count decreased at higher antibiotic concentrations. In contrast, the GenR strain showed no significant changes in OMV production but exhibited an increase in particle size (148.5 nm compared to 127 nm in the control). In response to treatment with 8 µg/mL of amoxicillin/clavulanate, the vesicular increase was 6- and 4.3-fold for GenS and GenR, respectively. Under iron-deficient conditions, induced by the chelator 2,2′-bipyridyl, both strains showed reduced growth, with a 30–33% decrease at 400 µM and a 51–53% decrease at 600 µM, but without significant changes in OMV production. However, the proteomic analysis of OMVs revealed substantial changes in protein composition. In the GenS strain, exposed to gentamicin, the number of proteins was 2–6 times higher than in the GenR strain. Under low-iron conditions, the GenR strain showed at least a 2.9-fold increase in protein concerning gentamicin exposure, while the GenS strain showed a 1.4-fold increase compared to antibiotic conditions. Overall, protein upregulation was more pronounced in the GenS strain, with a significant increase in stress proteins, such as ClpB, HscA, Lon, and ClpP. In contrast, proteome upregulation in GenR was less pronounced, with ClpP upregulated similarly under both low-iron and gentamicin exposure, though without a concurrent increase in chaperone proteins. These results highlighted the crucial role of OMVs in the *E. coli* survival response to environmental stresses, emphasizing their potential impact on disease progression and antibiotic resistance dynamics [151]. Other studies have used *E. coli* as a model to investigate not only OMV production but also the amount of associated Stx2a toxin in enterohemorrhagic *E. coli* (EHEC) strains O104:H4 and O157:H7. The exposure of these strains to ciprofloxacin resulted in a significant increase in OMV production 250-fold for *E. coli* O104:H4 and 183-fold for O157:H7. Similarly, the amount of Stx2a associated with OMVs increased 143-fold for O104:H4 and 123-fold for O157:H7, while OMV cytotoxicity increased 1024-fold and 512-fold, respectively. Comparable effects were observed with mitomycin C, which increased OMV production 568-fold for O104:H4 and 470-fold for O157:H7 and raised OMV-associated Stx2a levels 332-fold and 275-fold, respectively. OMV cytotoxicity also increased in both strains. In contrast, antibiotics such as fosfomycin, meropenem, and polymyxin B increased OMV production without affecting the amount of OMV-associated Stx2a or cytotoxicity. Fosfomycin increased OMV production 77-fold for O104:H4 and 24-fold for O157:H7, meropenem by 27-fold for O104:H4 and 14-fold for O157:H7, and polymyxin B by 9-fold for O104:H4 and 7-fold for O157:H7. However, these antibiotics did not affect OMV-associated Stx2a levels or cytotoxicity. Gentamicin, rifaximin, tigecycline, and azithromycin did not influence OMV production in either strain but modulated the amount of OMV-associated Stx2a and cytotoxicity differently. Specifically, rifaximin, tigecycline, and azithromycin significantly reduced Stx2a levels and OMV cytotoxicity in the O104:H4 strain but did not impact the O157:H7 strain. Chloramphenicol reduced both cytotoxicity and Stx2a levels in OMVs in both strains without altering OMV production. The findings suggest that the increase in OMV production depended on the antibiotic’s mechanism of action. Ciprofloxacin and mitomycin C activate the SOS response, promoting the release of OMVs and Stx2a. In contrast, fosfomycin, meropenem, and polymyxin B act as cell membrane stressors, stimulating OMV production without increasing Stx2a levels. This behavior underscored the role of OMVs as a stress-response mechanism in bacteria. The study also emphasized the importance of avoiding ciprofloxacin in EHEC infections, as its strong induction of Stx2a-associated OMVs could increase the risk of hemolytic–uremic syndrome. Even fosfomycin, which did not increase Stx2a levels, could worsen clinical outcomes by increasing OMV production and triggering inflammatory processes. Conversely, protein synthesis inhibitors such as rifaximin and azithromycin, which did not increase OMV or Stx2a production, could represent safer treatment options [152]. Recent data have expanded the understanding of the effects of antibiotics on OMV production. These findings have confirmed that antibiotics can also influence the presence of resistance genes in OMVs, thus contributing to the risk of resistance spread. The study investigated the effects of antibiotics on tigecycline-resistant *E. coli* (47EC). The strain was resistant to tigecycline (MIC: 8 µg/mL), chloramphenicol (MIC: 64 µg/mL), and rifaximin (MIC: >256 µg/mL), and susceptible to ceftazidime (MIC: ≤1 µg/mL), ciprofloxacin (MIC: ≤0.25 µg/mL), polymyxin B (MIC: ≤0.5 µg/mL), and meropenem (MIC: ≤0.25 µg/mL). Exposure to antibiotics at sub-inhibitory concentrations significantly increased OMV production. The protein concentration of OMVs without antibiotics was 0.709 ± 0.03 mg/mL, while with antibiotics it ranged from 0.768 ± 0.02 mg/mL (ceftazidime ½ × MIC) to 6.333 ± 0.15 mg/mL (mitomycin C ¼ × MIC). Ciprofloxacin induced a dose-dependent increase, reaching 4.938 ± 1.19 mg/mL at ½ × MIC and 6.03 ± 0.90 mg/mL at ¼ × MIC. The mean size of OMVs without antibiotics was 140.5 nm, whereas mitomycin C and ciprofloxacin increased to approximately 178.3–268.7 nm. Exposure to tigecycline, meropenem, gentamicin, and rifaximin (½ × MIC) produced smaller OMVs (89.2–137.4 nm). The zeta potential of untreated OMVs was −5.057 ± 0.956, whereas with some antibiotics, it decreased to values ranging from −14.725 ± 1.366 (mitomycin C) to −5.6 ± 2.229 (rifaximin). The *tet* (X4) gene associated with tigecycline resistance was present in OMV, with increased amounts in OMV treated with all antibiotics except rifaximin and tigecycline. A transcriptomic analysis with ciprofloxacin identified 386 genes with altered transcription, including those associated with the SOS response and DNA repair. Quantitative RT-PCR confirmed significant variations in the expression of genes *recA* (17.64-fold), *cho* (3.31-fold), *lexA* (5.68-fold), *dcrB* (2.72-fold), and *RS14425* (185.29-fold), suggesting that ciprofloxacin stimulated OMV production through DNA damage and the activation of the SOS response. In conclusion, antibiotics stimulated OMV production in resistant bacteria, increasing the risk of HGT [153].

#### 2.1.3. OMVs Response to Antibiotics in Other Gram-Negative Bacteria

Antibiotic exposure can influence OMV secretion in other Gram-negative bacteria, with effects depending on the antibiotic type and bacterial strain. Under antibiotic stress, these bacteria often release an increased number of OMVs, likely as a defensive mechanism to protect the bacterial cell. These vesicles contain proteins that contribute to antibiotic resistance, potentially altering the surrounding environment and facilitating bacterial adaptation to antimicrobial treatments. For instance, Yun and colleagues analyzed the OMVs of *Acinetobacter baumannii* DU202 using a proteogenomic approach. They found that approximately 35.5% of membrane proteins were present in OMVs, indicating selective enrichment during vesicle formation. Notably, prophage gene clusters and their protein products constituted a significant portion of the OMVs. Imipenem treatment reduced the expression of phage proteins by 40% while increasing the expression of β-lactamase OXA-23. In imipenem-treated OMVs (OMV-IM), OXA-23 represented 36% of the total proteins, marking a 9.23-fold increase compared to untreated OMVs (OMV-LB). A differential protein expression analysis revealed an increase in serine proteases and M23 family peptidases in OMV-IM. Furthermore, the vesicles were enriched in OMPs, including OmpA and OmpW, implicated in virulence, biofilm formation, and antibiotic resistance. These findings suggested that OMVs modified by imipenem treatment served as carriers of virulence factors and antibiotic resistance determinants [154]. The ability of OMVs to encapsulate virulence agents has also been studied in *Shigella dysenteriae* type 1, which has altered the production and release of Shiga toxin under antimicrobial influence. Shiga toxin, associated with hemorrhagic colitis, hemolytic uremic syndrome, and thrombotic thrombocytopenic purpura, was studied in three bacterial strains treated with five antimicrobials, including mitomycin C and fosfomycin. Mitomycin C significantly increased Shiga toxin production and release in OMVs. After 24–48 h of incubation at 37 °C, toxin concentrations in OMVs were significantly higher than those in the supernatant or bacterial pellet. In contrast, ciprofloxacin, norfloxacin, fosfomycin, and nalidixic acid did not significantly affect toxin production or release. Electron microscopy revealed that treatment with mitomycin C (150 µL/mL) increased the number and size of OMVs (20–150 nm in diameter), which were highly electron-dense at the center. Conversely, cultures treated with other antibiotics or untreated exhibited fewer and smaller vesicles. These results demonstrated that mitomycin C was a potent inducer of Shiga toxin production and release, with OMVs serving as the primary vehicles for toxin transport and accumulation [155]. *Klebsiella pneumoniae* (*K. pneumoniae*) OMVs have also been studied for their ability to respond to antibiotic stress by carrying resistance and virulence factors. The KpHCD1 strain was exposed to subinhibitory concentrations of meropenem, amikacin, polymyxin B, and their combination. The MIC values were 128 mg/mL for meropenem, 16 mg/mL for amikacin, and 32 mg/mL for polymyxin B, confirming that KpHCD1 was resistant to these drugs. Subinhibitory concentrations for OMV production were set at 25% and 10% of the MIC values for each antibiotic. Despite variations in particle concentrations among the conditions, OMVs had an average size of approximately 150 nm across all treatments. Exposure to meropenem (32 mg/mL) and polymyxin B (6.4 mg/mL) resulted in an approximately two-fold increase in OMV production compared to the untreated strain. In contrast, treatment with amikacin (4 mg/mL) reduced OMV production to approximately half the level observed in the untreated strain. The combined use of meropenem, amikacin, and polymyxin B did not result in a significant increase in OMV production compared to antibiotics alone. The proteomic analysis of OMVs revealed significant antibiotic-induced changes in protein expression, with 72 proteins identified in control OMVs, 363 in meropenem-treated OMVs, 138 in amikacin-treated OMVs, 393 in polymyxin B-treated OMVs, and 220 in OMVs from the combined antibiotic condition. Among these, 57 proteins were common across all conditions, while others were condition specific. A gene ontology analysis indicated enrichment in proteins associated with the cell membrane, translation, and macromolecular complexes. Additionally, 15 antibiotic resistance genes and 27 virulence factors were identified, including OMPs, iron-binding factors, and efflux pumps. A differential expression analysis revealed that antibiotic treatments altered the expression of proteins involved in genetic information processing, energy metabolism, and drug resistance. For instance, polymyxin B upregulated proteins related to energy metabolism, while meropenem affected proteins involved in cell division and cell wall synthesis. Efflux pump AcrB was upregulated in response to polymyxin B and meropenem, underscoring KpHCD1’s capacity to modulate protein-level responses to antibiotic-induced stress and enhance the synthesis of virulence factors [156]. Some researchers have hypothesized that the morpho-functional modulation of OMVs following exposure to stressors was a protective mechanism employed by the bacterium itself. A study by Murray and colleagues emphasized the role of Helicobacter pylori (H. pylori) OMVs in protecting against various immune stressors and antibiotics. The addition of OMVs restored H. pylori survival after treatment with 1 mM H_2_O_2_, which alone resulted in a reduction of more than 8 logarithmic units in bacterial colony counts. This effect was attributed to catalase present in the vesicular lumen. Furthermore, OMVs mitigated bacterial growth inhibition in the presence of antimicrobial peptides. The peptide LL-37 inhibited H. pylori growth within a concentration range of 1.25–5 µg/mL. However, 50 µg/mL OMVs enhanced bacterial growth at all doses tested, with the maximal protective effect observed at 5 µg/mL of LL-37. It is hypothesized that this protection may result from the sequestration of the peptide by OMVs or from the release of inhibitors following vesicular lysis. Furthermore, OMVs did not show protection against amoxicillin and metronidazole, suggesting that they were not able to interfere with the action of this antibiotic. Against clarithromycin and levofloxacin, the protective effect was dose-dependent, even after heat treatment at 80 °C, indicating that the effect was not mediated by thermosensitive enzymes. Considering the lipophilic nature of these antibiotics, the authors hypothesized that OMV acted by sequestering them in the vesicular lumen and thus preventing entry into bacterial cells. These findings underscore the essential role of OMVs as a survival mechanism for H. pylori under oxidative stress conditions and in the presence of specific antibiotics [157]. Similarly, *Stenotrophomonas maltophilia* (*S. maltophilia*) has been shown to respond to antibiotic stress through protective mechanisms. This bacterium is recognized as an emerging MDR organism, with an increasing prevalence in cystic fibrosis patients. Key findings have highlighted the production of OMVs under the influence of imipenem and QS molecules, such as DSF (cis-Δ2-11-methyl-dodecenoic acid). This study revealed increased vesicle secretion and the expression of two vesicle-associated proteins homologous to Ax21 (Smlt0387 and Smlt0184). Moreover, OMVs produced in the presence of imipenem contained elevated amounts of β-lactamases L1 and L2, suggesting that this is part of the bacterium’s defense strategy against antibiotic stress. Regarding the impact of QS molecules on OMV production, DSF produced by *S. maltophilia* led to an increase in OMV secretion comparable to that induced by imipenem, while BDSF (cis-Δ2-dodecenoic acid), produced by *Burkholderia cenocepacia*, induced a more modest increase. In contrast, PDSF (cis-Δ2-decenoic acid), produced by *P. aeruginosa,* had no effect, confirming that *S. maltophilia* is unable to perceive this signal. Overall, the study demonstrated that this strain employed OMVs as an adaptive strategy to antibiotic stress and in cellular communication dynamics [158]. A recent study has highlighted that this modulatory mechanism not only affected pathogenic bacteria but also environmental bacteria. Altering the latter’s responses could compromise their ecological role, potentially disturbing the ecosystem balance and environmental sustainability. Fang and colleagues investigated the impact of antibiotics at sub-inhibitory concentrations (sub-MIC) on the Geobacter sulfurreducens-OMVs, a bacterium known for its ability to reduce iron oxides via extracellular electron transfer. This process, crucial for the biogeochemical cycling of iron, carbon, nitrogen, sulfur, and phosphorus, has significant applications in the bioremediation of organic pollutants and heavy metals. Exposure to sub-inhibitory concentrations (1/4 1/8, 1/16, and 1/32 × MIC) of ampicillin and ciprofloxacin resulted in a significant increase in OMV production, with ampicillin inducing a 1.075- to 2.4-fold increase and ciprofloxacin a 1.4- to 5.6-fold increase, compared to controls. Antibiotic-induced OMVs showed similar sizes, but TEM images revealed distinct morphologies, with denser clusters and double-layered structures, particularly in the case of ciprofloxacin, suggesting the formation of outer–inner OMVs (OIMVs) through explosive cell lysis. Antibiotic-treated OMVs demonstrated a greater ability to reduce Fe (III) and exchange electrons than control OMVs. A UV-Vis analysis revealed a higher content of cytochromes in antibiotic-induced OMVs, with a marked enrichment of cytochromes in ciprofloxacin-OMVs. Moreover, a prote analysis identified a greater number of proteins in antibiotic-induced OMVs, with an increased abundance of cytochrome c, especially in ciprofloxacin-OMVs. A total of 734,918, and 1343 proteins were identified in control, ciprofloxacin-induced, and ampicillin-induced OMVs, respectively. Proteins associated with the SOS response and DNA repair were more abundant in ciprofloxacin-OMVs, suggesting phage activation. The analysis also indicated that ampicillin stimulates the production of OMVs through OM swelling, whereas ciprofloxacin induces the formation of OIMVs via cell lysis, mediated by the SOS response and prophage activation. Proteins such as OmpA and those of the Tol-Pal system, involved in membrane integrity, were negatively regulated in antibiotic-induced OMVs, highlighting distinct mechanisms in the biogenesis of ampicillin- and ciprofloxacin-induced OMVs [159].

### 2.2. Antibiotic-Induced Modulation of MVs in Gram-Positive Bacteria

MVs produced by Gram-positive bacteria have emerged as a critical research focus for understanding microbial adaptation mechanisms and host–pathogen interactions. Despite their significance, studies on these systems remain scarce, with limited exploration of their biogenesis, composition, and functional roles. This section will report the evidence available on the modulation of MVs in Gram-positive bacteria under antibiotic stress, highlighting how these conditions influence their production and biological properties (Table 2).

One of the pioneering studies examined the production of MVs by *Enterococcus faecium* (*E. faecium*) ATCC 700221 cultured with and without antibiotics and evaluated their pathological effects on human epithelial cells. Bacteria were grown in BHI broth to the late exponential phase, and MVs were isolated from the culture supernatant. The TEM analysis revealed that *E. faecium* produced spherical MVs smaller than 50 nm, while the SDS-PAGE analysis highlighted distinct protein profiles between bacterial lysates and MVs. The strain was resistant to vancomycin (MIC 512 µg/mL) but susceptible to linezolid (MIC 2 µg/mL). Exposure to subinhibitory concentrations of vancomycin (256 µg/mL) and linezolid (1 µg/mL) increased MV-associated protein production by 3- and 1.5-fold, respectively, compared to growth in BHI alone. Protein concentrations increased from 65.0 ± 3.2 µg/L to 197.0 ± 5.4 µg/L with vancomycin and to 95.8 ± 3.9 µg/L with linezolid. The proteomic analysis identified 438 proteins in MVs from BHI cultures, including 265 cytoplasmic, 153 membrane, and 20 extracellular proteins. MVs from vancomycin- and linezolid-exposed cultures contained 461 and 513 proteins, respectively, with 301 shared across all conditions. Unique proteins were identified in each group: 51 in BHI-derived MVs, 56 in vancomycin-derived MVs, and 76 in linezolid-derived MVs. In human Caco-2 epithelial cells, MVs produced in the presence of linezolid exhibited higher cytotoxicity than those from vancomycin-treated or untreated cultures. Cytotoxic effects were observed at concentrations ≥ 5 µg/mL for MVs/BHI and MVs/VAN and 0.5 µg/mL for MVs/LIN. Flow cytometry confirmed the greater cytotoxicity of MVs/LIN. Regarding inflammatory potential, MVs/BHI significantly upregulated IL-1β, IL-6, IL-8, and MCP-1 expression but not TNF-α. MVs/LIN elevated IL-1β, IL-6, and IL-8 levels while reducing TNF-α expression. Conversely, MVs/VAN elicited a stronger pro-inflammatory response, increasing IL-1β, IL-6 and TNF-α expression, with less pronounced effects on IL-8 and MCP-1. Treatment with proteinase K demonstrated that degradable proteins contributed to inflammatory responses but not significantly to cytotoxicity, suggesting a role for non-protein components or proteinase K-resistant proteins in cytotoxic effects. These findings were pivotal in unraveling the complexity of MVs, including peptidoglycans and other pathogen-associated molecular patterns (PAMPs) mediating interactions with host cells. Furthermore, *E. faecium* MVs acted as potent virulence factors, thus influencing therapeutic outcomes and host–pathogen dynamics [163]. In the same year, Andreoni and colleagues investigated the MVs role produced by *Staphylococcus aureus* (*S. aureus)* under stress conditions, emphasizing the molecular pathways regulating their formation and protective functions. Antibiotics and DNA-damaging agents, such as mitomycin C, were shown to stimulate MVs production through two primary mechanisms: (i) a phage-dependent pathway, linked to the SOS response and endolysin-induced cell lysis; and (ii) a phage-independent pathway, mediated by cell wall damage. Under standard conditions, prophage-bearing *S. aureus* strains (NRS77phage and RN4220phage) exhibited no significant differences in MV production compared to their prophage-free counterparts (NRS135 and RN4220). However, exposure to subinhibitory mitomycin C concentrations induced a concentration-dependent increase in MV formation in lysogenic strains, whereas prophage-free strains did not exhibit a similar response. A TEM analysis detected MVs and phages in mitomycin C treated lysogenic strains, supporting the role of prophage-induced cell lysis as a central mechanism in MV biogenesis. Ciprofloxacin at ¼ × MIC-value significantly increased MV production in lysogenic strains, while no comparable effect was observed in prophage-free strains. In contrast, β-lactam antibiotics such as flucloxacillin and ceftaroline, administered at 10 × MIC, stimulated MVs production through a phage-independent mechanism, with less pronounced effects at ¼ × MIC. Electron microscopy confirmed that flucloxacillin-generated MVs lack phages, suggesting an alternative formation mechanism linked to cell wall structural integrity. A DNA content analysis revealed that MVs generated via prophage-induced cell lysis contain higher DNA levels than those produced through a blebbing mechanism, potentially due to bacterial chromosome fragmentation during the lytic cycle. Furthermore, flucloxacillin-MV derivatives demonstrated a dose-dependent protective role for *S. aureus* against daptomycin. This protective capacity was confirmed by incubating flucloxacillin-MVs with *S. aureus* for 30 min, before exposure to daptomycin. Overall, this study underscored how antibiotic-induced MV production may hinder the eradication of *S. aureus* during infections, suggesting a critical role for MVs in promoting bacterial persistence [161]. Considering the same bacterial species, another study explored the MVs role secreted by methicillin-resistant *S. aureus* (MRSA) in conferring resistance to β-lactam antibiotics. Vesicles were isolated from bacteria cultured under normal conditions (MVNor) and in the presence of sub-lethal concentrations of ampicillin (MVStrs). A TEM analysis confirmed that both vesicle types exhibited a spherical bilayer structure, while DLS measurements indicated a mean diameter of 78.22 ± 0.81 nm for MVStrs and 86.84 ± 0.25 nm for MVNor. The polydispersity index (PDI) was below 0.3 for both, indicating a monodisperse distribution. A zeta potential of approximately -30 mV suggested comparable structural stability. Exposure to increasing concentrations of ampicillin significantly enhanced MV production. At 64 μg/mL ampicillin, MVStrs secretion was 22.4-fold higher than MVNor. Notably, MVStrs degraded up to 640 μg/mL ampicillin within 3 h, whereas MVNor failed to degrade this dose even after 6 h. The proteomic analysis identified 159 proteins in MVStrs, many linked to β-lactam resistance, including β-lactamases encoded by the *blaZ* gene and penicillin-binding proteins (PBP1, PBP2, and PBP3). β-lactamase activity was up to 19.1-fold higher in MVStrs compared to MVNor. Functional assays demonstrated that MVStrs enabled the growth of *S. aureus* ATCC 29213 even in the presence of β-lactam antibiotics above the MIC. However, MVStrs did not confer protection against non β-lactam antibiotics, such as chloramphenicol, gentamicin, and tetracycline. Of note, MVs did not induce permanent genetic mutations in susceptible bacteria; the observed protection was exclusively mediated by molecules encapsulated within the MVs. Furthermore, MVs from MRSA failed to protect Gram-negative bacteria, including *E. coli*, *Edwardsiella tarda*, and *Salmonella* spp., underscoring the species-specific nature of this protective mechanism. These findings revealed that ampicillin stress enhanced MV production and altered their protective and degradative capacities against antibiotics, underscoring the pivotal role of MV-associated proteins in bacterial resistance. Thus, bacterial responses to antibiotic stress modulated the production and composition of MVs, influencing virulence, antibiotic resistance, and host–pathogen interactions. Understanding these mechanisms is critical for developing innovative therapeutic strategies [162].

### 2.3. Major Enzymes and Protein Classes Involved in OMV-Mediated Antibiotic Resistance

As previously outlined in this review, the significant contribution of OMV and MV to microbial antibiotic resistance is reduced through several strategies. In this section, we will provide an outline of the major protein classes found in OMVs produced from resistant strains, which are involved in their antibiotic resistance potential. These proteins mainly encompass all different β-lactamases and carbapenemase enzymes, membrane protein transporters, and efflux pumps.

#### 2.3.1. β-Lactamases and Carbapenemases

β-lactam antibiotics, cephalosporins, and carbapenems have been the most common antibiotics used over the last decades. Consequently, this is the class of antibiotics against which bacterial resistance has mainly developed. Therefore, β-lactamases and carbapenemases, antibiotic-degrading enzymes, have significantly evolved in different variants and are widely diffused among resistant strains. Their genes are present in most resistance plasmids spread among microbial populations [163]. The β-Lactamases are divided into four main classes based on their molecular mechanism, substrate specificity and evolution (A, B, C, and D). Class A, C, and D β-Lactamases are Serine β-Lactamases, while class D β-Lactamases are Metallo-lactamases. More in-depth, it is worth mentioning that some enzyme classes that evolved the capacity to hydrolyze several types of β-lactams: (i) The extended-spectrum β-Lactamases (ESBLs) are class A enzymes and an evolution of the narrow-spectrum β-Lactamases, which can hydrolyze a great variety of β-lactam antibiotics; and (ii) the serine-type carbapenemases, which are also class A and can hydrolyze almost all β-lactams. In this group, the most spread enzymes are those derived from *K. pneumoniae*, the *K. pneumoniae* carbapenemase (KPC); (iii) the AmpC β-Lactamases, which are class C enzymes able to hydrolyze the third-generation cephalosporins like cefotaxime and ceftazidime and are not inhibited by clavulanic acid; and (iv) the OXA-type carbapenemases, class D enzymes derived from *A. baumannii*. These are also serine-type β-lactamases that hydrolyze several third-generation penicillins, cephalosporins, and carbapenems. Finally, v) the NDM β-lactamases (New Delhi Metallo-β-lactamase), are class B enzymes and are frequently membrane-bound. These are the ones with the broadest spectrum, even though they require Zn^2+^ for their activity [164]. Enzymes belonging to all these groups have also been retrieved in OMVs produced by resistant strains.

*K. pneumoniae* is one of the most common pathogens endowed with wide-spectrum antibiotic resistance, and all classes of β-lactamases are expressed by this bacterium. In the OMVs isolated from different *K. pneumoniae* carbapenem-resistant strains (CRKP), Yao and coworkers documented the presence of bla-KPC and bla-NDM, activities endowed with the ability of hydrolyzing meropenem. They reported the epidemiological distribution of these enzymes in almost 150 CPKP isolates, with the bla-KPC being the most abundant. Importantly, the KPC enzyme appeared to be protected and highly stable within the OMV, resisting degradation unless the vesicle membrane was disrupted [165]. Moreover, the authors highlighted the role of OMVs in resistance by both shielding the producing strain by direct antibiotic degradation and protecting neighboring susceptible strains through KPC extracellular trafficking. In several cases, it was observed that OMV-mediated mobilization of resistance enzymes spreads resistance also among different species. For example, OMVs derived from *K. pneumoniae* and loaded with KPC conferred resistance to *P. aeruginosa* against the carbapenem antibiotic imipenem [166]. The study provides strong experimental evidence that KPC-loaded OMVs not only protect susceptible *P. aeruginosa* strains from imipenem-induced killing but also promote the emergence of resistant subpopulations. In vitro, the addition of KPC-OMVs to susceptible *P. aeruginosa* allowed survival under imipenem pressure. This protective effect was confirmed in vivo using a *Galleria mellonella* infection model, where OMV-treated larvae exhibited decreased mortality despite imipenem administration. However, a key finding of the study was that *P. aeruginosa* did not acquire the bla-KPC gene from OMVs, but low OMV concentrations led to sub-MIC environments, favoring spontaneous mutations that confer resistance. The *P. aeruginosa* carbapenem-resistant subpopulations emerged, all of which had mutations in the OprD porin gene, for which no mutation was carried in OMVs.

Many other papers describe the OMV-mediated resistance transfer in other bacterial species. Martínez and coworkers explore the role of *E. coli*-OMVs in disseminating New Delhi metallo-β-lactamase 1 (NDM-1), a critical carbapenemase contributing to antimicrobial resistance [167]. The authors provide compelling in vivo evidence that OMVs act as delivery systems for active NDM-1, enhancing cross-species protection against carbapenem antibiotics, particularly meropenem. NDM-1 is a Zn(II)-dependent β-lactamase uniquely localized to the OM due to its lipoprotein nature. This membrane anchoring facilitates its selective secretion into OMVs as an active enzyme. Using *G. mellonella* larvae as an infection model, the authors demonstrate that OMVs encapsulating NDM-1 confer robust protection to otherwise carbapenem-susceptible *E. coli* and *P. aeruginosa* strains. The OMVs not only extend the enzyme stability in the host but also outperform free soluble NDM-1 in conferring resistance. The study rules out HGT as the main mechanism of resistance dissemination in this context by using plasmid incompatibility controls and differential antibiotic resistance markers. Instead, it highlights OMVs as efficient protein-based vectors of resistance, supporting bacterial community survival during antibiotic treatment.

Other studies document the broad versatility of OMVs in conferring either transient or stable antibiotic resistance in different bacterial strains, through enzyme delivery. Some additional examples encompass *Salmonella enterica* and *Neisseria gonorrhoeae*, for which evidence was presented that OMVs fuse with susceptible bacteria, efficiently delivering β-lactamase into recipient cells [168,169]. As already briefly outlined, for their intrinsic nanolipidic structure, OMVs are particularly suited to deliver membrane-bound proteins and macromolecules. In this context, NDM carbapenemases are often enriched in the membrane of OMVs from resistant strains, thus boosting NDM diffusion in the microenvironment and enhancing its stability during the delivery. However, NDMs are not the only membrane-bound resistance enzymes found in OMVs. In this context, Capodimonte and coworkers recently described the secretion of membrane-anchored Class D OXA β-lactamases from *A. baumannii* in OMVs [170]. The authors described the presence of a lipobox motif in the signal peptides of these enzymes that is critical for their lipidation and membrane anchoring, enabling their incorporation into OMVs. Unlike OXA-48 from Enterobacterales, which lacks a lipobox and remains soluble in the periplasm, clinically important OXAs such as OXA-23 and OXA-24/40 in *A. baumannii* are anchored to the OM and actively secreted via OMVs. Functional assays showed that OMVs enriched with lipidated OXAs confer significant protection against piperacillin and imipenem to otherwise susceptible bacterial strains (e.g., *E. coli*, *P. aeruginosa*). In contrast, OMVs loaded with soluble forms of OXA enzymes provided reduced protective capacity. This evidence highlighted the importance of membrane localization in resistance propagation. The study also raises the possibility that such OMV-mediated processes are under-recognized in clinical settings but represent a powerful evolutionary strategy for the dissemination of carbapenem resistance and warrant further investigation. Altogether, these findings suggest that OMVs are not passive carriers of β-lactamases enzymes but functionally relevant particles that disseminate resistance determinants in polymicrobial environments.

Noteworthily, β-lactamases resistance is spread through OMVs also by the horizontal transfer of non-plasmid genetic material. Innovative works investigate the role of OMVs in conferring resistance through the horizontal transfer of antibiotic resistance genes (ARGs), encoded by whole chromosomal clusters. In this context, Xu et al. assessed OMVs as transfer vectors of 11 non-plasmid ARGs, clustered in a 25-kb resistance island, from the pathogen *Avibacterium paragallinarum* to a non-pathogen homologous strain, obtaining the resistance acquisition of this latter [171]. However, despite the confirmation of ARGs in transformants, a lack of integration and no significant increase in MIC were observed after transformation, indicating non-persistence of the transferred ARGs across passages. The same trend was also demonstrated in *K. pneumonia* [172]. These studies confirmed that OMVs could act as vehicles for the horizontal transfer of non-plasmid beta-lactamase genes; the efficiency and stability of this transfer remain, however limited, suggesting that OMVs may contribute to resistance spread but are less effective than plasmid-based HGT in maintaining resistance over time.

#### 2.3.2. Efflux Pumps

Recent studies underscore the importance of the OM environment remodeling, once the antibacterial response is triggered. A central role is played by membrane transporters, porins, and efflux pumps, which are the main regulators of cellular trafficking with the environment. Efflux pumps—particularly those belonging to the resistance–nodulation–division (RND) superfamily, such as AdeABC, AcrAB-TolC, and MexAB-OprM—have been recurrently identified within the proteomic cargo of OMVs derived from antibiotic-stressed pathogens, including *A. baumannii*, *E. coli*, *K. pneumoniae*, and *P. aeruginosa* [173,174,175,176]. These tripartite systems, capable of spanning the bacterial envelope, are not only overexpressed in response to antimicrobial pressure but are also selectively incorporated into OMVs, suggesting a strategic vesicle-mediated extrusion of toxic compounds and intercellular dissemination of resistance determinants [174,176,177]. Functionally, such vesicles have been shown to confer transient resistance to susceptible bacteria and modulate the periplasmic and extracellular antibiotic milieu. For instance, OMVs from wild-type *P. aeruginosa* containing MexAB-OprM significantly enhanced ciprofloxacin tolerance in recipient cells, while vesicles from efflux-deficient mutants did not [177]. Similarly, *A. baumannii* exposed to tetracycline displayed the upregulation of AdeABC components in OMVs, reinforcing the hypothesis that vesicle cargo is adaptively restructured under stress [178]. Beyond the RND systems, pumps from the major facilitator superfamily (MFS) such as NorA and MepA in *S. aureus* and AcrA/B in Enterobacteriaceae also appear to contribute to OMV-based resistance, either by direct extrusion or by influencing vesicle biogenesis pathways [173,177,179]. Although the precise mechanisms governing the sorting and activity of efflux pumps in OMVs remain to be fully elucidated, their consistent detection across multiple pathogens and experimental models supports a conserved and evolutionary favorable strategy. This integration of membrane-bound efflux machinery into extracellular compartments highlights OMVs as not merely passive blebs but as active players in bacterial adaptation to antibiotic pressure, presenting novel targets for efflux pump inhibition and OMV interference strategies [180,181].

#### 2.3.3. OMVs Proteome Remodeling

In this final section, some hints are provided of studies that mainly focused on investigating how antibiotic treatment affects the proteomic landscape of OMVs. Polymyxin B treatment in extensively drug-resistant *K. pneumoniae* led to significant changes in the OMV proteome, showing alterations in proteins involved in β-lactam resistance, cationic antimicrobial peptide resistance, LPS biosynthesis and modification, peptidoglycan biosynthesis, and efflux systems [182]. Apart from β-lactames, the key functional categories affected are the following:

- Cationic antimicrobial peptide (CAMP) resistance proteins, for example, 4-amino-4-deoxy-L-arabinose transferase and PhoPQ two-component kinase that allows lipid A and OM modifications and are required for resistance to polymyxin and AMPs. Other related enzymes are undecaprenyl phosphate-alpha-L-Ara4N transferase, copper homeostasis protein, and thiol: disulfide interchange protein;
- OM and envelope remodeling, such as proteins for LPS biosynthesis and O-antigen modification (e.g., KdsA, GmhA, and HldE). The OmpA and others (e.g., OmpX and asmA) showed significant reduction in polymyxin-treated susceptible OMVs;
- Two-component systems (TCS) CpxA, a protein involved in envelope stress response, is downregulated following the treatment;
- Protein export and translocation systems, significant changes in Sec and Tat pathways are observed (e.g., the downregulation of SecA, YajC, and YidC), and the downregulation of SurA, a key chaperone for OM protein folding;
- RNA processing and repair: altered abundance of RNA degradosome proteins (e.g., RNase E, RhlB, and GroEL) and nucleotide excision repair proteins were observed upon polymyxin treatment.

The findings provide insights into how specific protein cargoes are selectively packaged into OMVs and how these may be exploited for resistance and survival. In conclusion, OMVs can help bacteria develop antibiotic resistance by both spreading resistance genes and proteins and acting as protective armors. The characterization of the enzymes and proteins involved in OMVs remodeling during antibiotic treatment may provide invaluable insights to understand the resistance mechanisms and strategies put in place by bacteria. These could be conjugated as direct antibiotic degradation, to complex OM remodeling with reduced influx and protection from antimicrobial intake. However, it is important to note that bacteria employ different strategies that are often specific to a particular antibiotic. This suggests that applying one resistance mechanism could also sensitize bacteria to another antibiotic class. The various studies described here contribute to the depiction of OMVs as key elements in the formation of bacterial communities and the spread of antibiotic resistance.

## 3. Conclusions

EVs represent a sophisticated bacterial adaptation mechanism capable of modulating responses to environmental pressures, including antibiotic exposure [183]. Antibiotic exposure induces an increase in EV secretion, accompanied by structural and biochemical modifications that enhance bacterial protective impact [162]. These vesicles serve as defense tools, detoxifying the surrounding environment through antibiotic incorporation to protect bacteria from the direct effects of antimicrobial molecules and facilitate intercellular communication to coordinate collective survival strategies [184,185]. In Gram-negative bacteria, OMVs undergo modifications in response to antibiotics, promoting HGT and reducing drug efficacy by sequestering antibiotics within the vesicular lumen [186]. In Gram-positive bacteria, MVs transport hydrolytic enzymes and other resistance factors, protecting bacterial cells and promoting collective survival [187,188]. A key aspect of EV function is their role in intercellular communication, coordinating adaptive responses such as QS and modulation of the host immune response [189]. Some EVs act as molecular traps for antibiotics, reducing their active concentration and limiting their effectiveness [190]. These findings have multiple clinical implications. Understanding the impact of antibiotics influencing EV secretion and function could pave the way for targeted therapeutic strategies [191]. Interfering with EV biogenesis may offer an innovative approach to limiting the spread of antibiotic resistance and reducing bacterial virulence [192]. At the same time, EV engineering could be leveraged to develop new drug delivery systems, vaccines, and alternative therapeutic strategies [193,194]. Advancing our understanding of these mechanisms could provide new opportunities to counteract antibiotic resistance and improve strategies for controlling bacterial infections.

## Figures and Tables

**Table 1:** Effects of antibiotics on the production, morphology, and composition of OMVs secreted by Gram-negative bacteria.

Bacteria	Antibiotic	Evidence	Reference
*P. aeruginosa*	Gentamicin	Gentamicin significantly increased the production of OMVs in *P. aeruginosa* by destabilizing the OM, enriching it with proteins, enzymes, and DNA, suggesting a role for OMVs in the transport of virulence factors during infection.	[147]
*P. aeruginosa*	Ciprofloxacin	Ciprofloxacin induces a significant increase in OMV production in *P. aeruginosa* through the activation of the SOS response, regulated by LexA, enriching the vesicles with proteins involved in antibiotic resistance and virulence, increasing the cytotoxicity and pathogenicity of the bacterium.	[148]
*P. aeruginosa*	Meropenem Ciprofloxacin	Combination therapy with meropenem and ciprofloxacin in MDR *P. aeruginosa* significantly reduces OMV production, attenuates bacterial virulence, and modulates the expression of genes associated with resistance and cellular repair, suggesting a potential benefit in the combined use of these antibiotics.	[149]
*P. aeruginosa*	Polymyxin B	*P. aeruginosa* OMVs regulate intercellular communication by influencing QS and biofilm formation, modulating polymyxin B resistance.	[150]
*E. coli*	Gentamicin Amoxicillin/ clavulanate	Exposure to gentamicin and amoxicillin/clavulanate modulates the production and protein composition of OMVs secreted by extraintestinal pathogenic *E. coli* strains.	[151]
*E. coli*	Ciprofloxacin Mitomycin C Fosfomycin Meropenem Polymixin B Rifaximin Tigecycline Azithromycin	Ciprofloxacin and mitomycin C increased OMVs in *E. coli* EHEC, with an increase in Stx2a toxin and cytotoxicity, whereas fosfomycin, meropenem, and polymyxin B stimulated only OMVs production. Rifaximin, tigecycline, and azithromycin reduced Stx2a and cytotoxicity.	[152]
*E. coli*	Ciprofloxacin Mitomycin C Ceftazidime Tigecycline Meropenem Gentamicin Rifaximin	Ciprofloxacin, mitomycin C, ceftazidime, tigecycline, meropenem, gentamicin, and rifaximin stimulated the production of OMVs in tigecycline-resistant *E. coli*, modifying their size and composition and promoting the spread of the *tet (X4)* resistance gene.	[153]
*A. baumanni*	Imipenem	Imipenem treatment reduced phage proteins but increased β-lactamase OXA-23, enriching *A. baumanni* OMVs with proteases and membrane proteins (OmpA, OmpW) associated with virulence, biofilm, and antibiotic resistance.	[154]
*S. dysenteriae*	Mitomycin C Ciprofloxacin, Norfloxacin Fosfomycin Nalidixic acid	Mitomycin C increased Shiga toxin production in OMVs in *S. dysenteriae* type 1, whereas ciprofloxacin, norfloxacin, fosfomycin, and nalidixic acid showed no significant effects. OMVs treated with mitomycin C were more numerous, larger (20–150 nm), and highly dense.	[155]
*K. pneumoniae*	Meropenem Polymyxin B	In *K. pneumoniae* KpHCD1, meropenem and polymyxin B increased OMV production, while amikacin decreased it. OMVs contained resistance genes and virulence factors, with protein expression modulated by antibiotics.	[156]
*H. pylori*	Clarithromycin Amoxicillin Metronidazole.	OMVs protected *H. pylori* bacteria from antimicrobial peptides and some antibiotics. OMVs sequestered clarithromycin, reducing its efficacy, but did not protect against amoxicillin or metronidazole.	[157]
*S. maltophilia*	Imipenem	Imipenem and QS molecules (DSF) increased OMVs production in *S. maltophilia*, enriching them with β-lactamases L1 and L2 to counteract antibiotic stress.	[158]
*G. sulfurreducens*	Ampicillin Ciprofloxacin	Ampicillin and ciprofloxacin increased the production of OMVs in *G. sulfurreducens*, altering their morphology and functionality. Ciprofloxacin induced OMVs through explosive cell lysis and phage activation, while ampicillin stimulated the formation of classical OMVs.	[159]

**Table 2:** Effects of antibiotics on the production, morphology and composition of Gram-positive bacteria MVs.

Bacteria	Antibiotic	Evidence	Reference
*E. faecium*	Vancomycin Linezolid	Vancomycin and linezolid increased MV production in *E. faecium* and altered their protein composition. MVs produced with linezolid showed greater cytotoxicity, while those produced with vancomycin induced a stronger inflammatory response.	[160]
*S. aureus*	Mitomycin C Ciprofloxacin Flucloxacillin Ceftaroline Daptomycin	Mitomycin C and ciprofloxacin stimulated MV production in *S. aureus* through a phage-dependent SOS response mechanism, whereas flucloxacillin and ceftaroline acted independently of phages, damaging the cell wall. The produced MVs protected from daptomycin treatment.	[161]
*S. aureus*	Ampicillin	Ampicillin increased the production of MVs derived from *S. aureus* MRSA, enriching them with β-lactamases and resistance-related proteins, allowing the degradation of the antibiotic and protecting *S. aureus* without transmitting genetic resistance.	[162]
